# Testosterone ameliorates age-related brain mitochondrial dysfunction

**DOI:** 10.18632/aging.203153

**Published:** 2021-06-17

**Authors:** Wensheng Yan, Tianyun Zhang, Yunxiao Kang, Guoliang Zhang, Xiaoming Ji, Xu Feng, Geming Shi

**Affiliations:** 1 Department of Neurobiology, Hebei Medical University, Shijiazhuang, China; 2 Neuroscience Research Center, Hebei Medical University, Shijiazhuang, China; 3 Hebei Laboratory Animal Center, Hebei Medical University, Shijiazhuang, China; 4 Hebei Key Laboratory of Forensic Medicine, Department of Forensic Medicine, Hebei Medical University, Shijiazhuang, China

**Keywords:** testosterone, mitochondrial function, antioxidative capacity, mitochondrial biogenesis, aged male rats

## Abstract

Brain mitochondrial dysfunction and reduced testosterone levels are common features of aging in men. Although evidence suggests that the two phenomena are interrelated, it is unclear whether testosterone supplementation ameliorates mitochondrial dysfunction in the aging male brain. Here, we show that testosterone supplementation significantly alleviates exploratory behavioral deficits and oxidative damage in the substantia nigra and hippocampus of aging male rats. These effects were consistent with improved mitochondrial function, reflected by testosterone-induced increases in mitochondrial membrane potential (MMP), antioxidant enzyme (GSH-PX, catalase, and Mn-SOD) expression/activity, and mitochondrial respiratory complex activities in both brain regions. Furthermore, elevated PGC-1α, NRF-1, and TFAM expression (suggestive of enhanced mitochondrial biogenesis), increased citrate synthase activity, mtDNA copy number, and ND1, COX1, and ATP6 expression (indicative of increased mitochondrial content), as well as increased PINK1/Parkin and decreased P62 expression (suggesting mitophagy activation), were detected in the substantial nigra and hippocampus of aged male rats after testosterone supplementation. These findings suggest that testosterone supplementation may be a viable approach to ameliorating brain mitochondrial dysfunction and thus prevent or treat cognitive-behavioral deficits and neurodegenerative conditions associated with aging.

## INTRODUCTION

Mitochondrial dysfunction is a common feature of normal aging and is closely associated with the development of age-related neurodegenerative disease [1–3]. Altered mitochondrial recycling, resulting from abnormal biogenesis/mitophagy cycles, coupled to decreased antioxidant capacity and hence unmitigated reactive oxygen species (ROS) production, all result in impaired ATP synthesis and trigger energy deficits largely responsible for the progressive cellular dysfunction characteristic of aging [3, 4]. Besides impairing ATP production by compromising the normal function of the respiratory chain machinery, excessive ROS generation and the ensuing oxidative stress cause oxidative damage to proteins, lipids, and DNA, accelerating the aging phenotype [5, 6]. Thus, ensuring normal mitochondrial function is critical for delaying aging and reducing the risk of age-related neurodegenerative disease [4, 7, 8].

An age-dependent decrease in circulating testosterone levels has been reported in men [9, 10]. Interestingly, such decrease has been correlated with the development of age-related neurodegenerative diseases such as Alzheimer’s disease (AD) and Parkinson’s disease (PD) [11]. Although testosterone supplementation in male patients was shown to improve cognitive function in AD [12, 13], and to relieve motor and nonmotor symptoms in PD [14, 15], it remains unclear whether testosterone beneficially influences mitochondrial function in the aging brain. This possibility is indeed supported by animal studies that showed that orchiectomy-induced testosterone deficiency reduces mitochondrial function and increases oxidative damage in the substantia nigra (SN) and the hippocampus (HIPP) of adult male rats [16–18].

Notwithstanding, there is a paucity of research on the effects of testosterone supplementation on age-related mitochondrial dysfunction in the brain. To address this issue, we evaluated behavioral responses, assessed neuronal function and integrity, and conducted a comprehensive analysis of mitochondria-related parameters in selected brain regions of aged (24 months old) male rats supplemented with testosterone propionate (TP).

## RESULTS

### Analysis of serum testosterone and body weight in TP-supplemented aged male rats

**Figure 1:**
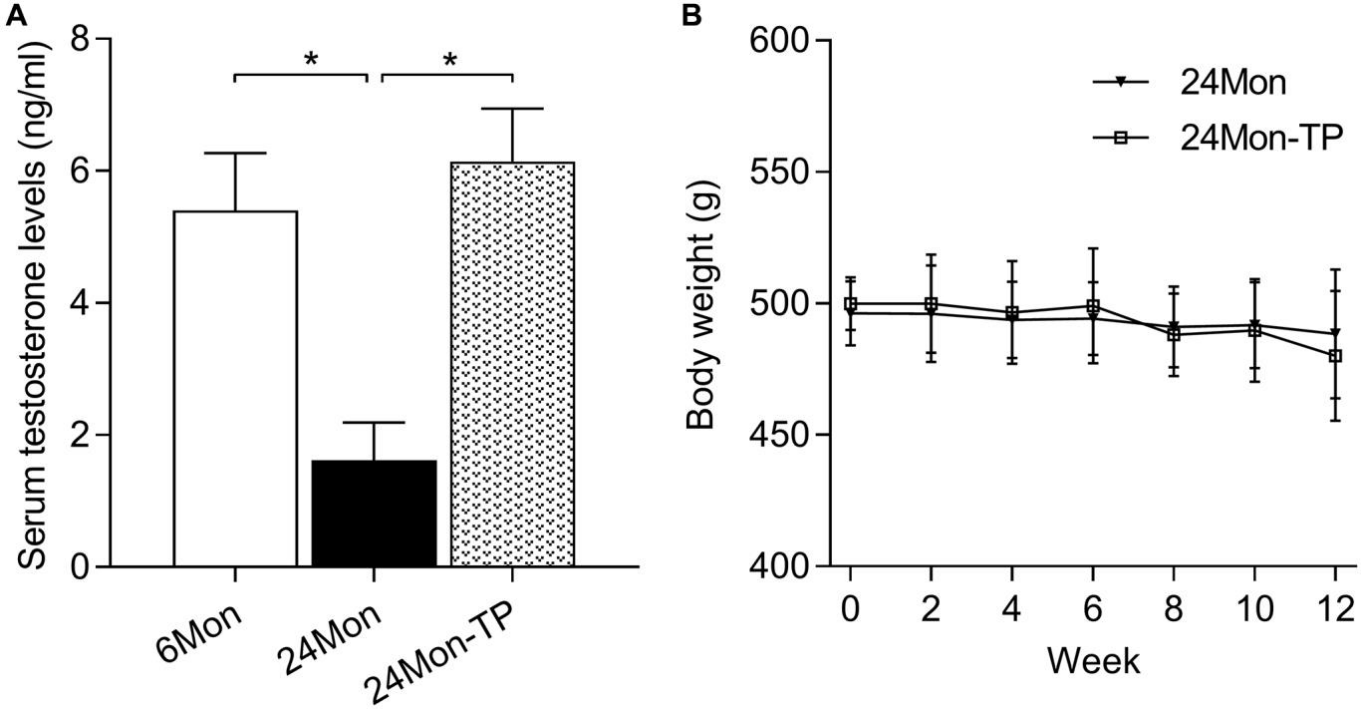
**Serum testosterone levels and body weight measurements.** (**A**) Serum testosterone levels recorded in the experimental animal groups. (**B**) Summary of body weight measurements. Data are expressed as the mean ± S.D. (*n* = 8/group). ^*^P < 0.01 (Bonferroni test).

The behavioral and brain-regional impact of testosterone supplementation was examined in 24-month-old male rats subjected to a 12-week TP injection regimen (24Mon-TP group). Untreated 6-month-old (6Mon) and vehicle-injected 24-month-old (24Mon) animals served as controls. Serum testosterone levels were significantly different among 6Mon, 24Mon, and 24Mon-TP rats (Figure 1A, F(2,21) = 82.787, *P* < 0.01). Specifically, lower testosterone levels were detected in 24Mon rats compared to both 6Mon and 24Mon-TP rats (*P* < 0.01), while no significant difference was observed between the last two groups. No significant changes in body weight were recorded during TP supplementation in 24Mon-TP compared to 24Mon rats (Figure 1B).

### TP supplementation alleviates exploratory behavioral deficits in aged male rats

**Figure 2:**
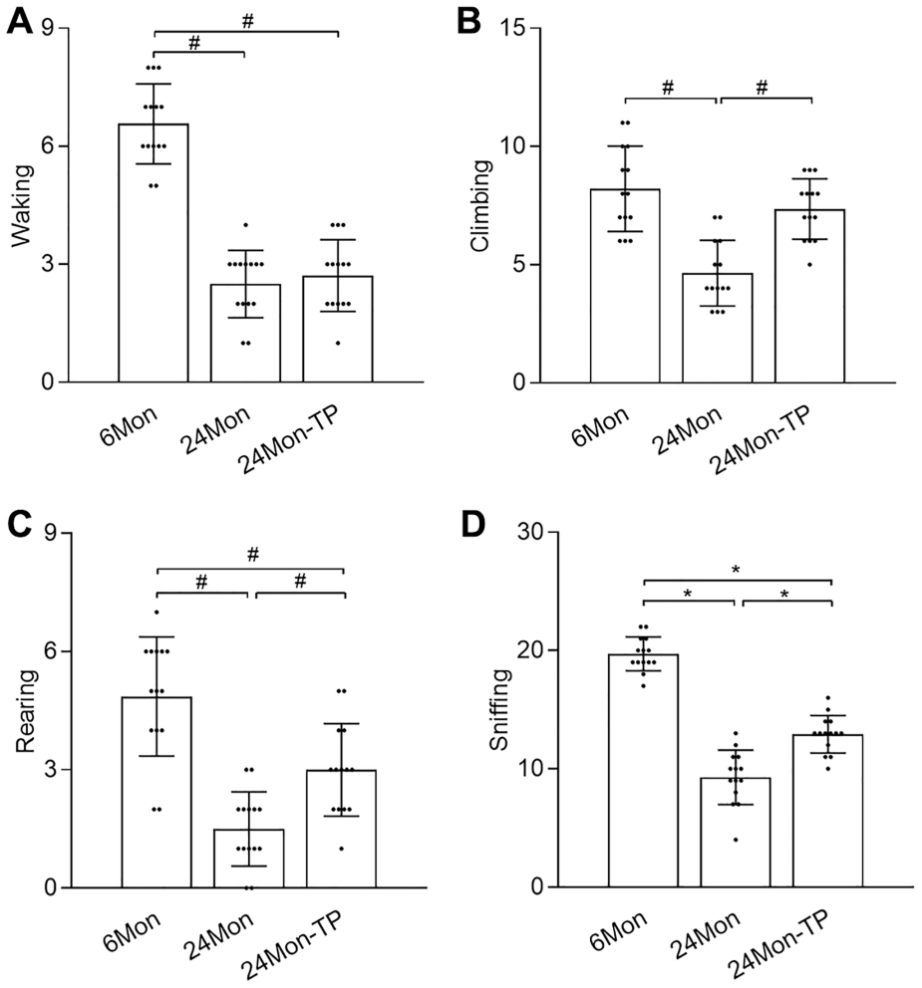
**TP supplementation ameliorates exploratory behavior deficits in aged male rats.** Open field test results for (**A**) walking, (**B**) climbing, (**C**) rearing, and (**D**) sniffing. Data are expressed as the mean ± S.D. (*n* = 14/group). ^*^P < 0.01 (Bonferroni test); ^#^*P* < 0.0167 (Mann-Whitney *U* test, Bonferroni correction).

The open field test was used to evaluate exploratory behavior in control and TP-supplemented rats. There were significant intergroup differences in walking (Figure 2A, χ^2^ = 30.519), climbing (Figure 2B, χ^2^ = 21.864), rearing (Figure 2C, χ^2^ = 28.537), and sniffing (Figure 2D, F(2,39) = 114.718, *P* < 0.01) behaviors. Post hoc testing showed that walking, climbing, rearing, and sniffing activities were significantly reduced in 24Mon compared to 6Mon rats. In contrast, compared to 24Mon rats significant increments in climbing (56.92%), rearing (100%), and sniffing (40.76%) behaviors were noted in 24Mon-TP animals. These data indicate that TP supplementation improves exploratory behavioral deficits in aged male rats.

### TP supplementation attenuates age-related neuronal dysfunction and neuronal loss

**Figure 3:**
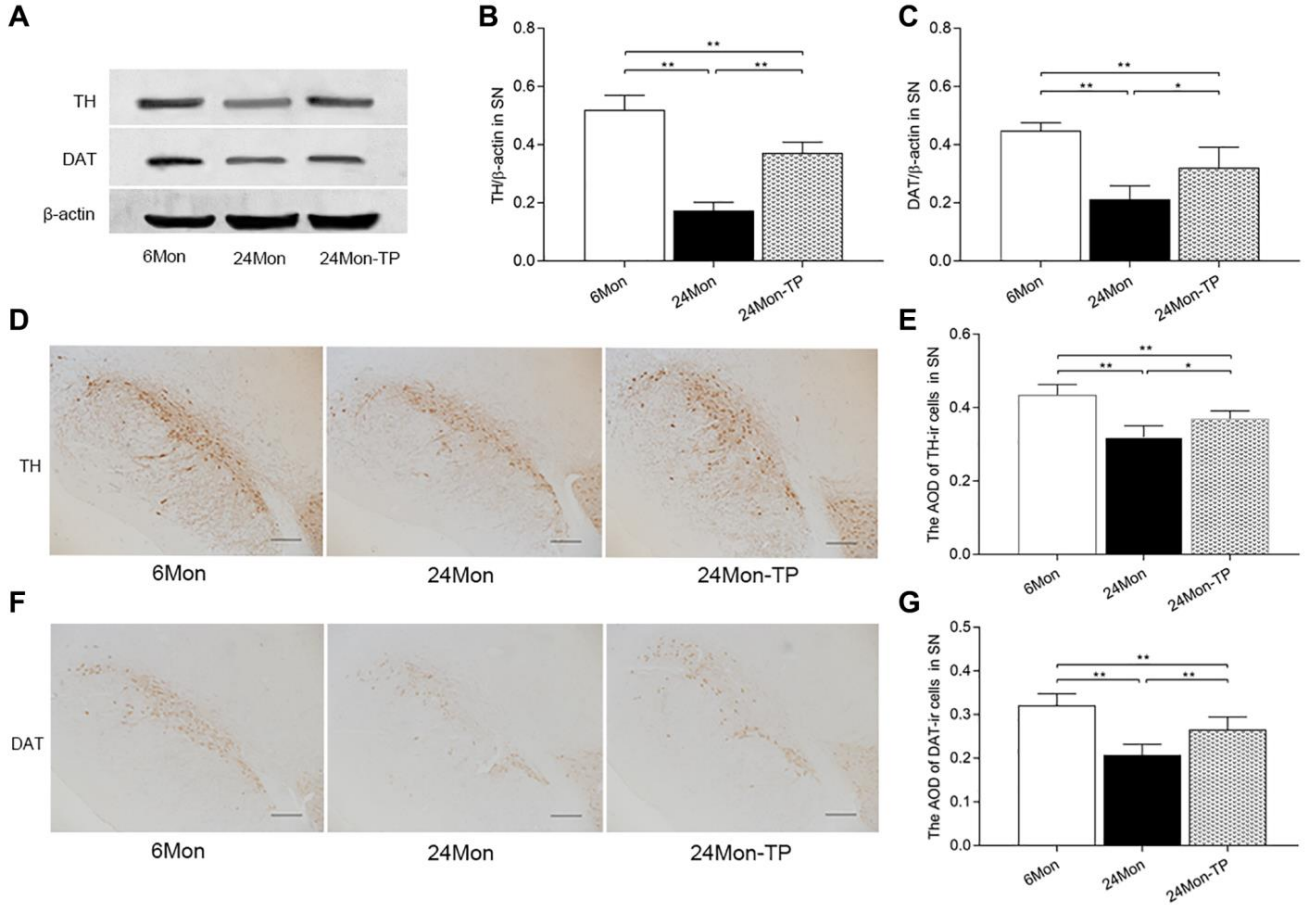
**TP supplementation increases the expression of TH and DAT in the SN of aged male rats.** (**A**) Representative western blots of TH and DAT expression. (**B**, **C**) Quantification of TH and DAT protein levels (normalized to β-actin; *n* = 5/group). (**D**–**G**) Representative IHC photomicrographs of TH and DAT expression in the SN of experimental rats (*n* = 6/group). Scale bars = 200 μm. Data are expressed as the mean ± S.D. ^*^P < 0.05, ^**^P < 0.01 (Bonferroni test).

The SN and the HIPP are two major brain structures controlling exploratory behavior [19, 20]. Thus, we analyzed neuronal status in these brain regions by detecting the expression of two markers of dopaminergic neurons, i.e., tyrosine hydroxylase (TH) and dopamine transporter (DAT), in the SN and by assessing the morphology of pyramidal cells in hippocampal CA1 area in the experimental rat groups. TH and DAT expression levels were lower in the 24Mon rats than 6Mon rats, but were significantly higher in 24Mon-TP rats than the 24Mon rats as revealed by western blot (Figure 3A–3C; TH, F(2,12) = 98.065; DAT, F(2,12) = 26.453; *P* < 0.01) and IHC (Figure 3D–3G; TH, F(2,15) = 26.731; DAT, F(2,15) = 27.336; *P* < 0.01).

**Figure 4:**
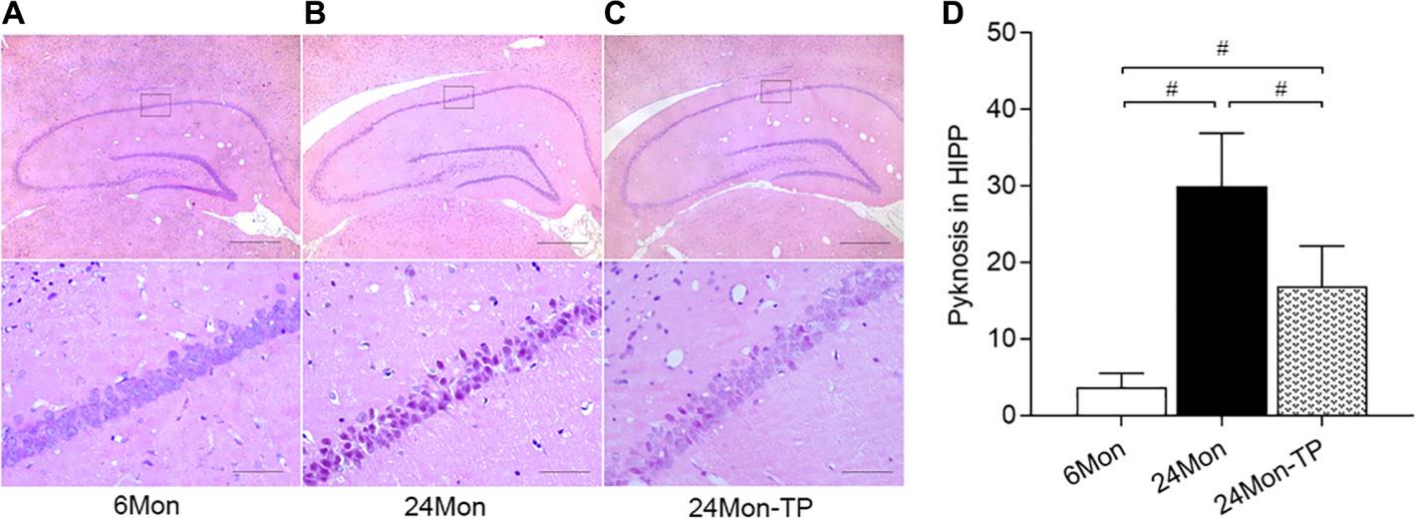
**TP supplementation preserves neuronal integrity in the HIPP of aged male rats.** (**A**–**C**) Representative photomicrographs of hippocampal sections stained with HE. Scale bars = 200 μm (upper panel), 20 μm (lower panel). (**D**) Quantification of karyopyknosis in the CA1 pyramidal stratum of the HIPP. Data are expressed as the mean ± S.D. (*n* = 6/group). ^#^*P* < 0.0167 (Mann-Whitney *U* test, Bonferroni correction).

While HE-stained neurons in the pyramidal stratum of the HIPP/CA1 area of 6Mon rats displayed large and clear nuclei (Figure 4A), obvious nuclear pyknosis was observed in a fraction of CA1 neurons in 24Mon rats (Figure 4B). However, compared to the latter group, CA1 samples from 24Mon-TP rats had much fewer pyknotic nuclei (Figure 4C). Intergroup variation in the number of cells with pyknotic appearance was significant (Figure 4D, χ^2^ = 14.407, *P* < 0.01). These findings demonstrate that TP supplementation attenuates neuronal dysfunction and neuronal loss in the SN and HIPP of aged male rats.

### TP supplementation increases mitochondrial membrane potential and mitochondrial respiratory complex activity in the aged male rat brain

**Figure 5:**
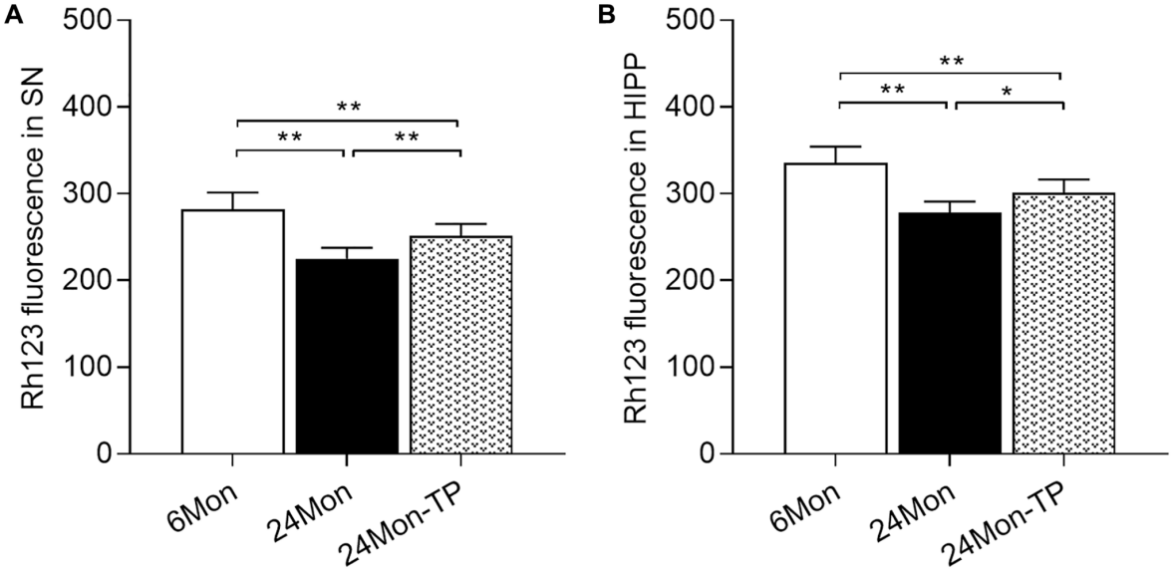
**TP supplementation increases mitochondrial membrane potential in the SN and HIPP of aged male rats.** MMP was detected by flow cytometry after labeling SN and HIPP cells with Rh123. (**A**) MMP analysis in dissociated SN cells. (**B**) MMP analysis in dissociated HIPP cells. Data are expressed as the mean ± S.D. (*n* = 8/group). ^*^P < 0.05, ^**^P < 0.01 (Bonferroni test).

**Figure 6:**
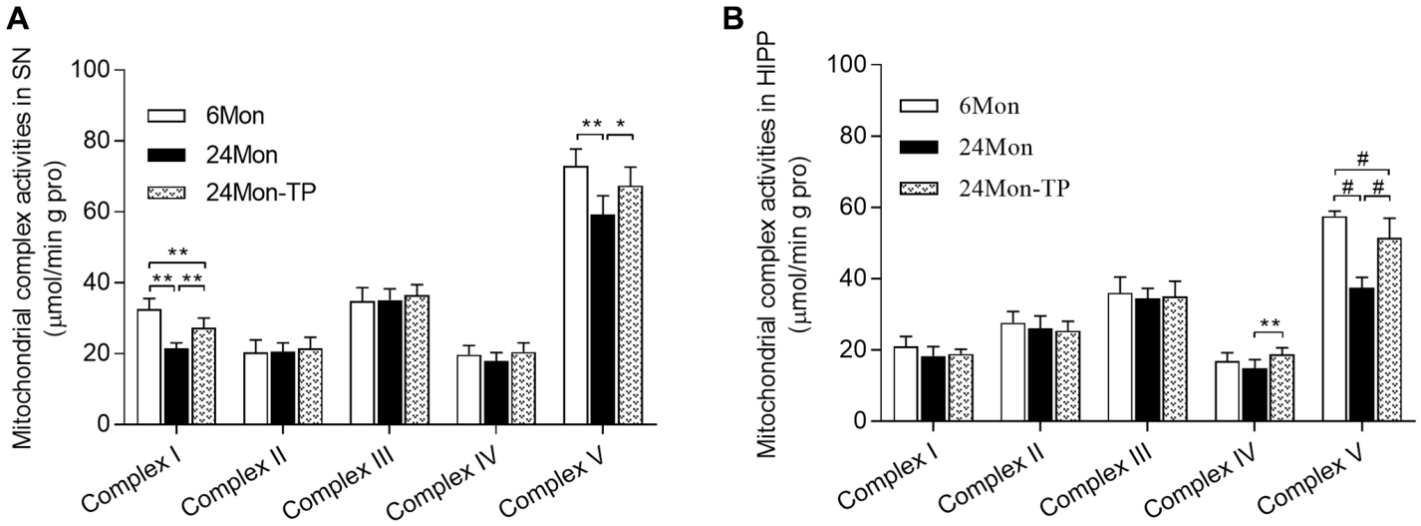
**TP supplementation stimulates mitochondrial respiratory complex activities in the SN and HIPP of aged male rats.** (**A**) Estimations of mitochondrial complexes I and V activities in the SN. (**B**) Estimations of mitochondrial complexes IV and V activities in the HIPP of aged male rats. Data are expressed as the mean ± S.D. (*n* = 8/group). ^*^P < 0.05, ^**^P < 0.01 (Bonferroni test); ^#^*P* < 0.0167 (Mann-Whitney *U* test, Bonferroni correction).

Mitochondrial dysfunction is intimately linked to brain energy deficit and neuronal dysfunction during aging [21, 22]. To evaluate whether TP supplementation can improve mitochondrial function in the aging brain, we analyzed mitochondrial membrane potential (MMP) and mitochondrial respiratory chain complex activity in the SN and HIPP of 6Mon, 24Mon, and 24Mon-TP rats. There were significant intergroup differences in MMP among cells of the SN (Figure 5A; F(2,21) = 27.134, *P* < 0.01) and the HIPP (Figure 5B; F(2,21) = 26.379, *P* < 0.01). MMP in the SN and HIPP was significantly decreased in 24Mon rats compared with 6Mon rats, and supplementation with TP increased MMP both in the SN (Figure 5A) and the HIPP (Figure 5B) in aged rats. In turn, significant intergroup differences were observed in the activities of mitochondrial complexes I and V in the SN (Figure 6A; Complex I, F(2,21) = 38.894; Complex V, F(2,21) = 14.627; *P* < 0.01) and complexes IV and V in the HIPP (Figure 6B; Complex IV, F(2,21) = 5.985; Complex V, χ^2^ = 18.24; *P* < 0.01). Complexes I and V activities in the SN, as well as complex V activity in the HIPP were reduced in 24Mon rats relative to 6Mon rats, and supplementation with TP increased complexes I and V activities in the SN (Figure 6A) and complexes IV and V activities in the HIPP (Figure 6B) in 24Mon-TP rats. Meanwhile, compared to 6Mon animals, the activities of mitochondrial complexes I and IV in the HIPP showed a slight, non-significant reduction in 24Mon control rats. These data suggest that TP supplementation alleviates mitochondrial dysfunction in the SN and HIPP of aged male rats.

### TP supplementation enhances antioxidative capacity in the aged male rat brain

**Figure 7:**
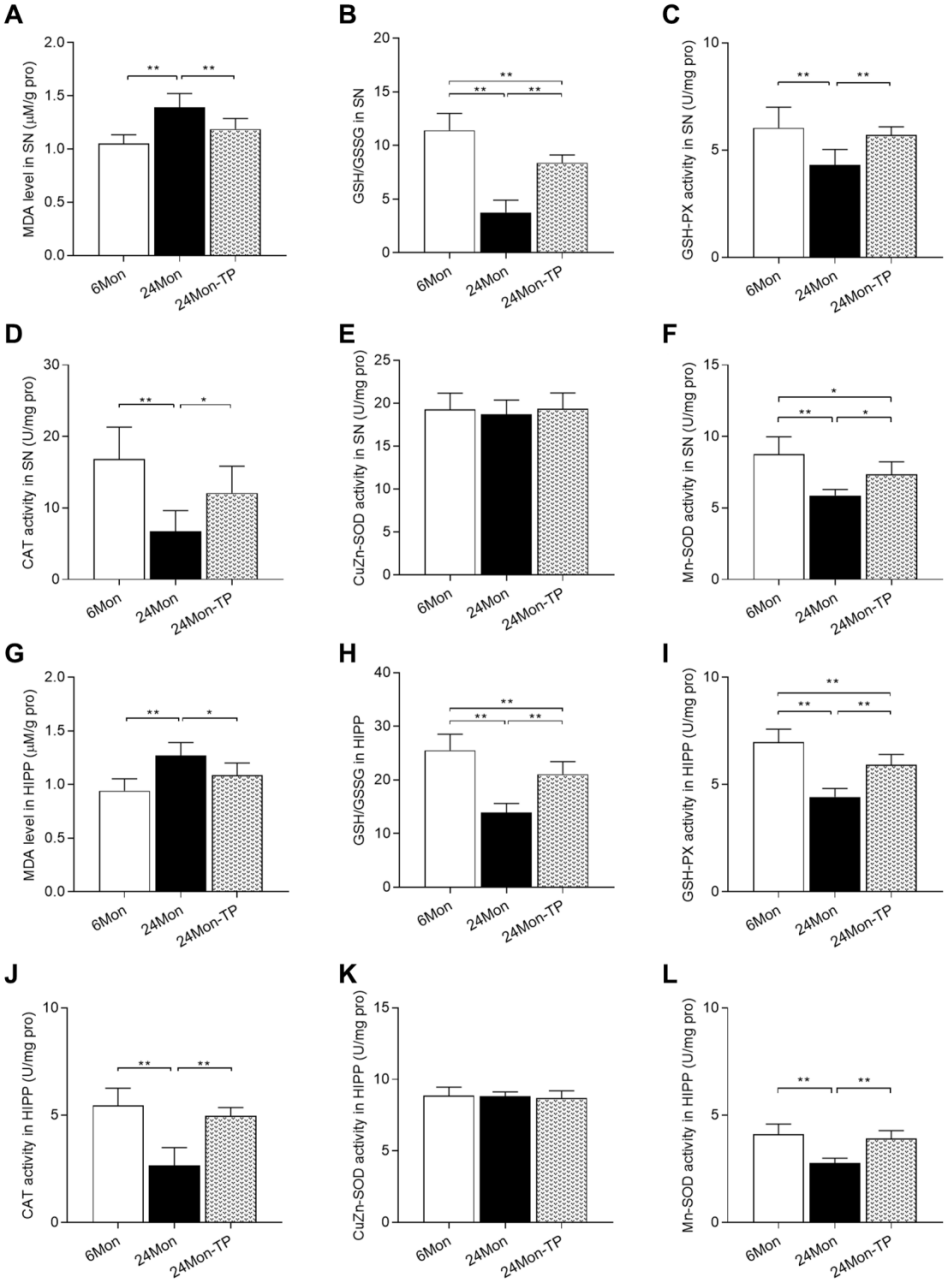
**TP supplementation enhances antioxidative potential in the SN and HIPP of aged male rats.** (**A**) MDA levels, (**B**) GSH/GSSG ratio, (**C**) GSH-PX activity, (**D**) CAT activity, (**E**) CuZn-SOD activity, and (**F**) Mn-SOD activity in the SN. (**G**) MDA levels, (**H**) GSH/GSSG ratio, (**I**) GSH-PX activity, (**J**) CAT activity, (**K**) CuZn-SOD activity, and (**L**) Mn-SOD activity in the HIPP. Data are expressed as the mean ± S.D. (*n* = 8/group). ^*^P < 0.05, ^**^P < 0.01 (Bonferroni test).

Since oxidative damage is critically involved in the aging process, we analyzed whether TP supplementation can improve antioxidative capacity in the brains of aged male rats by measuring SN/HIPP levels of malondialdehyde (MDA, a marker of ROS-mediated cell membrane damage), reduced glutathione/oxidized glutathione ratio (GSH/GSSG, a major biomarker of redox status in biological systems), as well as the antioxidant activities of glutathione peroxidase (GSH-PX), catalase (CAT), copper/zinc superoxide dismutase (CuZn-SOD), and manganese superoxide dismutase (Mn-SOD). Significant intergroup differences in MDA levels, GSH/GSSG ratio, and GSH-PX, CAT, and Mn-SOD activities were observed in the SN and HIPP among 6Mon, 24Mon, and 24Mon-TP rats (Figure 7A–7D and 7F; SN: MDA, F(2,21) = 21.181; GSH/GSSG, F(2,21) = 80.434; GSH-PX, F(2,21) = 12.595; CAT, F(2,21) = 14.509; Mn-SOD, F(2,21) = 20.369; *P* < 0.01. Figure 7G–7J and 7L; HIPP: MDA, F(2,21) = 16.132; GSH/GSSG, F(2,21) = 47.145; GSH-PX, F(2,21) = 52.537; CAT, F(2,21) = 35.595; Mn-SOD, F(2,21) = 30.132; *P* < 0.01). Post hoc testing revealed that MDA levels in the SN and HIPP were significantly increased in 24Mon rats compared with 6Mon rats, and supplementation with TP decreased MDA levels in both brain regions in aged rats (Figure 7A and 7G). The GSH/GSSG ratio, as well as the activities of GSH-PX, CAT, and Mn-SOD were all significantly decreased in the SN and HIPP of 24Mon rats compared with 6Mon rats, but were instead increased in 24Mon-TP rats relative to 24Mon rats (Figure 7B–7D, 7F, 7H–7J, and 7L). There was no difference in CuZn-SOD activity in the SN and HIPP among 6Mon, 24Mon, and 24Mon-TP rats (Figure 7E and 7K).

**Figure 8:**
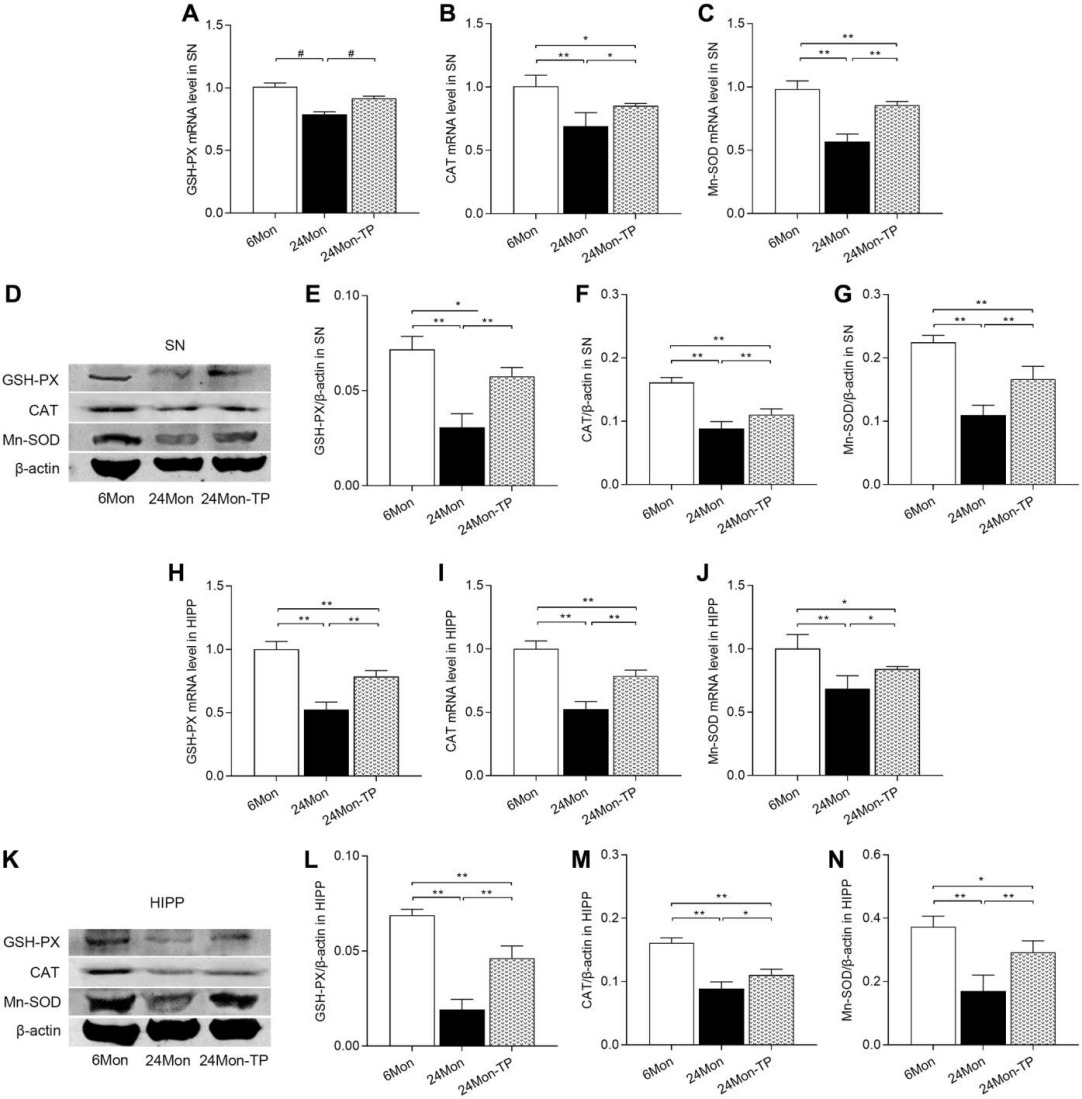
**TP supplementation increases antioxidant enzyme expression in the SN and HIPP of aged male rats.** (**A**–**C**) mRNA levels of GSH-PX, CAT, and Mn-SOD in the SN. (**D**) Representative western blots of GSH-PX, CAT, and Mn-SOD expression in the SN. (**E**–**G**) Quantification of GSH-PX, CAT, and Mn-SOD expression in the SN (normalized to β-actin). (**H**–**J**) mRNA levels of GSH-PX, CAT, and Mn-SOD in the HIPP. (**K**) Representative western blots of GSH-PX, CAT, and Mn-SOD expression in the HIPP. (**L**–**N**) Quantification of GSH-PX, CAT, and Mn-SOD expression in the HIPP (normalized to β-actin). Data are expressed as the mean ± S.D. (*n* = 5/group). ^*^P < 0.05, ^**^P < 0.01 (Bonferroni test); ^#^*P* < 0.0167 (Mann-Whitney *U* test, Bonferroni correction).

To confirm the antioxidant effects of TP supplementation on aged rat brains, we further measured GSH-PX, CAT, and Mn-SOD mRNA and protein levels in both brain regions. Significant intergroup differences in the expression of the three enzymes were observed for both mRNA expression (Figure 8A–8C; SN: GSH-PX, χ^2^ = 10.5; CAT, F(2,12) = 19.029; Mn-SOD, F(2,12) = 78.906; *P* < 0.01. Figure 8H–8J; HIPP: GSH-PX, F(2,12) = 90.185; CAT, F(2,12) = 92.034; Mn-SOD, F(2,12) = 16.07; *P* < 0.01) and protein levels (Figure 8D–8G; SN: GSH-PX, F(2,12) = 54.654; CAT, F(2,12) = 77.981; Mn-SOD, F(2,12) = 63.929; *P* < 0.01. Figure 8K–8N; HIPP: GSH-PX, F(2,12) = 115.144; CAT, F(2,12) = 83.126; Mn-SOD, F(2,12) = 31.289; *P* < 0.01). In turn, increased GSH-PX, CAT, and Mn-SOD mRNA/protein expression was detected in the SN and HIPP of 24Mon-TP rats relative to 24Mon rats. These data confirm that TP supplementation enhances antioxidative capacity in the SN and HIPP of aged male rats.

### TP supplementation promotes mitochondrial biogenesis in the aged male rat brain

**Figure 9:**
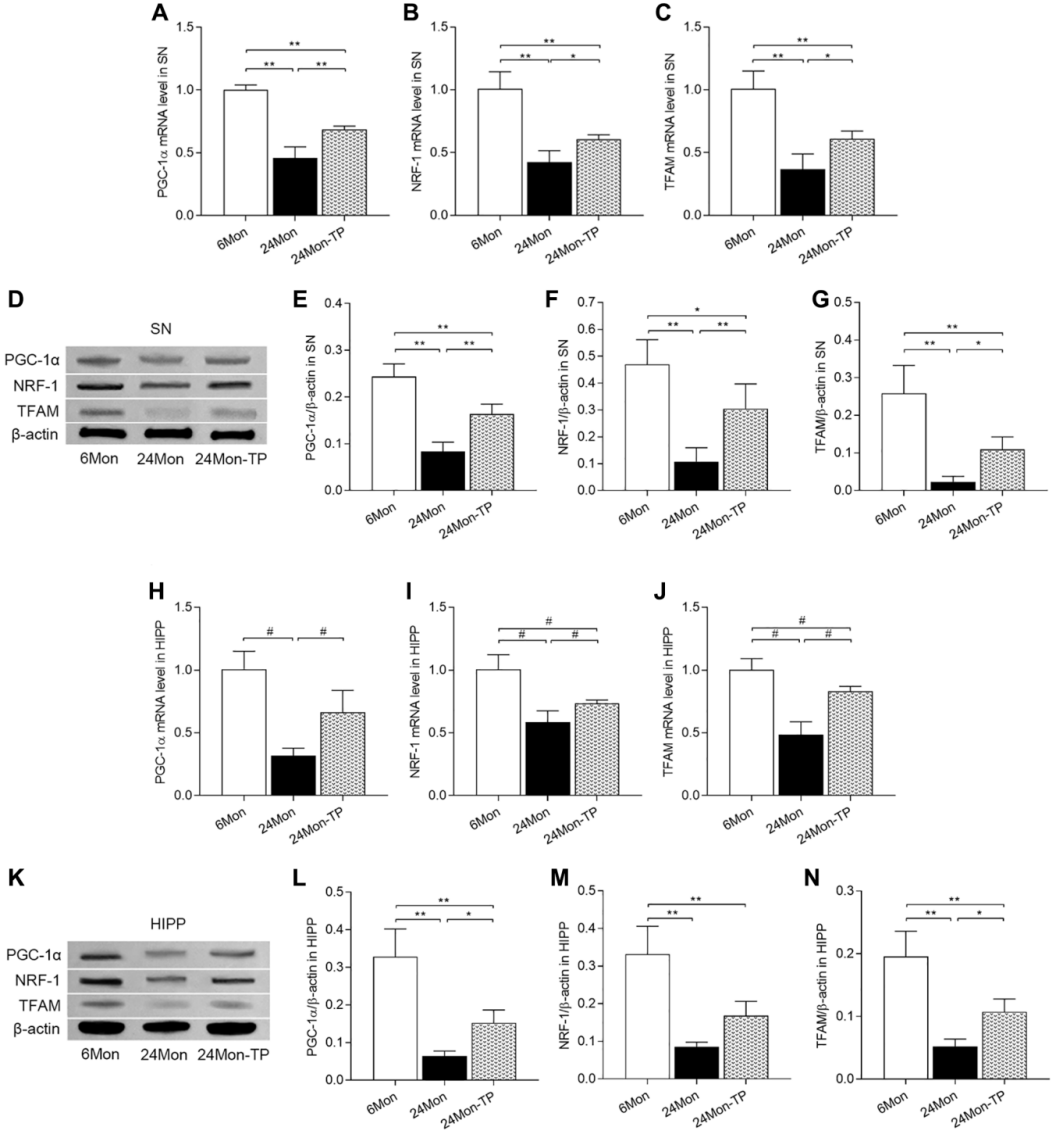
**TP supplementation promotes mitochondrial biogenesis in the SN and HIPP of aged male rats.** (**A**–**C**) mRNA levels of PGC-1α, NRF-1, and TFAM in the SN. (**D**) Representative western blots of PGC-1α, NRF-1, and TFAM expression in the SN. (**E**–**G**) Quantification of PGC-1α, NRF-1, and TFAM expression in the SN (normalized to β-actin). (**H**–**J**) mRNA levels of PGC-1α, NRF-1, and TFAM in the HIPP. (**K**) Representative western blots of PGC-1α, NRF-1 and TFAM expression in the hippocampus. (**L**–**N**) Quantification of PGC-1α, NRF-1, and TFAM expression in the HIPP (normalized to β-actin). Data are expressed as the mean ± S.D. (*n* = 5/group). ^*^P < 0.05, ^**^P < 0.01 (Bonferroni test); ^#^*P* < 0.0167 (Mann-Whitney *U* test, Bonferroni correction).

Based on the above findings indicating improved mitochondrial function and enhanced antioxidative potential in the brains of TP-supplemented aged male rats, we examined the effects of TP supplementation on the expression of key inducers and effectors of mitochondrial biogenesis, namely peroxisome proliferator-activated receptor gamma coactivator 1-alpha (PGC-1α), nuclear respiratory factor 1 (NRF-1), and mitochondrial transcription factor A (TFAM). In both SN and HIPP, significant differences among the experimental groups were detected for PGC-1α, NRF-1, and TFAM mRNA expression (Figure 9A–9C; SN: PGC-1α, F(2,12) = 109.146; NRF-1, F(2,12) = 47.166; TFAM, F(2,12) = 40.302; *P* < 0.01. Figure 9H–9J; HIPP: PGC-1α, χ^2^ = 12.02; NRF-1, χ^2^ = 12.5; TFAM, χ^2^ = 12.277; *P* < 0.01) and protein levels (Figure 9D–9G; SN: PGC-1α, F(2,12) = 61.105; NRF-1, F(2,12) = 25.386; TFAM, F(2,12) = 31.116; *P* < 0.01. Figure 9K–9N; HIPP: PGC-1α, F(2,12) = 39.891; NRF-1, F(2,12) = 33.152; TFAM, F(2,12) = 36.079; *P* < 0.01). PGC-1α, NRF-1, and TFAM mRNA and protein levels were significantly decreased in 24Mon rats relative to both 6Mon and 24Mon-TP rats (Figure 9A–9G for SN; Figure 9H–9N for HIPP). These findings suggest that TP supplementation to aging male rats promotes brain mitochondria biogenesis.

### TP supplementation increases mitochondrial content in the aged male rat brain

**Figure 10:**
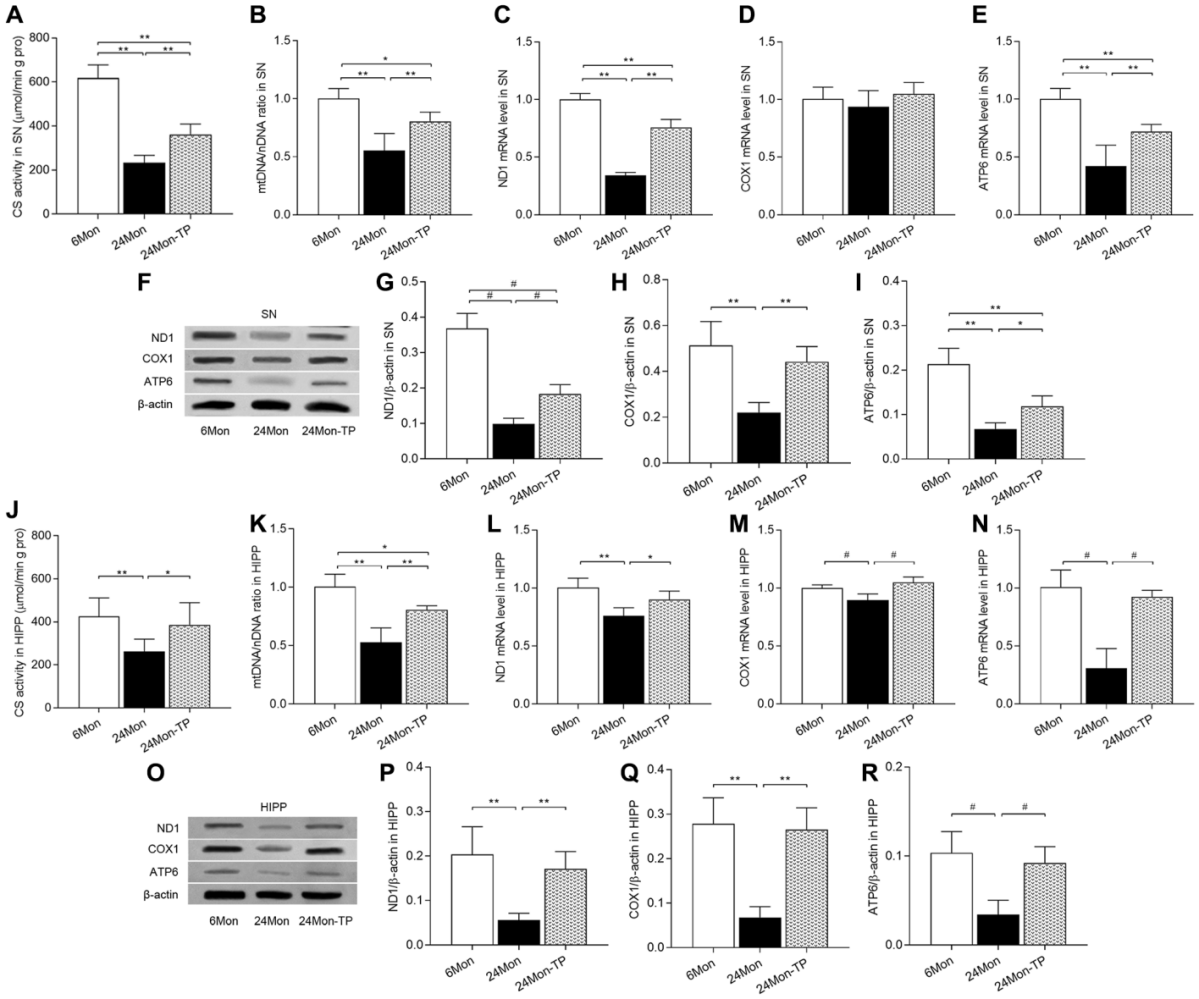
**TP supplementation increases mitochondrial content in the SN and HIPP of aged male rats.** (**A**) Citrate synthase activity in the SN. (**B**) mtDNA copy number in the SN. (**C**–**E**) mRNA levels of ND1, COX1, and ATP6 in the SN. (**F**) Representative western blots of ND1, COX1, and ATP6 expression in the SN. (**G**–**I**) Quantification of ND1, COX1, and ATP6 protein levels in the SN (normalized to β-actin). (**J**) Citrate synthase activity in the HIPP. (**K**) mtDNA copy number in the HIPP. (**L**–**N**) mRNA levels of ND1, COX1, and ATP6 in the HIPP. (**O**) Representative western blots of ND1, COX1, and ATP6 expression in the HIPP. (**P**–**R**) Quantification of ND1, COX1, and ATP6 expression in the HIPP (normalized to β-actin). Data are expressed as the mean ± S.D. (*n* = 5/group). ^*^P < 0.05, ^**^P < 0.01 (Bonferroni test); ^#^*P* < 0.0167 (Mann-Whitney *U* test, Bonferroni correction).

To verify that stimulation of mitochondrial biogenesis by TP supplementation is accompanied by increased mitochondrial contents, we measured the activity of citrate synthase (CS, a mitochondrial matrix enzyme), mitochondrial DNA (mtDNA) copy number, as well as expression levels of mtDNA-encoded subunits ND1, COX1, and ATP6 in the SN and HIPP of 6Mon, 24Mon, and 24Mon-TP rats. Significant intergroup differences were observed in CS activity (Figure 10A; SN, F(2,21) = 130.444; Figure 10J; HIPP, F(2,21) = 8.294; *P* < 0.01), mtDNA copy number (Figure 10B; SN, F(2,12) = 21.536; Figure 10K; HIPP, F(2,12) = 30.979; *P* < 0.01), and ND1, COX1 (except for SN, Figure 10D), and ATP6 mRNA (Figure 10C and 10E; SN: ND1, F(2,12) = 196.296; ATP6, F(2,12) = 28.378; *P* < 0.01. Figure 10L–10N; HIPP: ND1, F(2,12) = 12.647; COX1, χ^2^ = 10.562; ATP6, χ^2^ = 9.62; *P* < 0.01) and protein (Figure 10F–10I; SN: ND1, χ^2^ = 12.59; COX1, F(2,12) = 19.905; ATP6, F(2,12) = 38.995; *P* < 0.01. Figure 10O–10R; HIPP: ND1, F(2,12) = 15.572; COX1, F(2,12) = 32.44; ATP6, χ^2^ = 9.764; *P* < 0.01). Compared to 6Mon rats, CS and mtDNA copy number were respectively reduced by 62.26% and 44.9% in the SN and by 38.42% and 47.1% in the HIPP of 24Mon rats. In contrast, in 24Month-TP rats these variables were significantly increased in both brain regions (Figure 10A and 10B for SN, Figure 10J and 10K for HIPP). Likewise, significantly increased ND1, COX1, and ATP6 expression was detected in the SN (Figure 10C, 10E–10I) and HIPP (Figure 10L–10R) of 24Mon-TP rats relative to 24Mon controls. These data indicate that TP supplementation increases mitochondrial content in the aged male rat brain.

### TP supplementation activates the PINK1/Parkin pathway in the aged male rat brain

**Figure 11:**
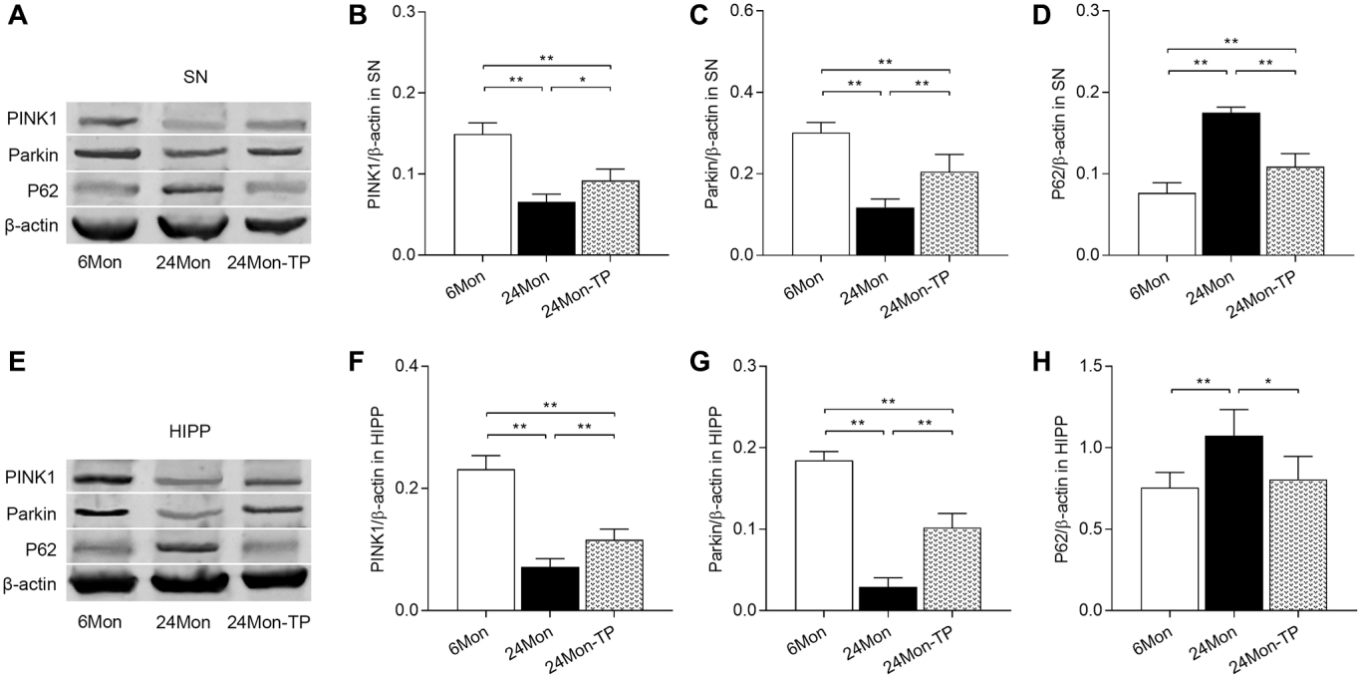
**TP supplementation activates PINK1/Parkin pathway in the SN and HIPP of aged male rats.** (**A**) Representative western blots of PINK1, Parkin, and P62 expression in the SN. (**B**–**D**) Quantification of PINK1, Parkin, and P62 of protein expression in the SN (normalized to β-actin). (**E**) Representative western blots of PINK1, Parkin, and P62 expression in the HIPP. (**F**–**H**) Quantification of PINK1, Parkin, and P62 protein levels in the HIPP (normalized to β-actin). Data are expressed as the mean ± S.D. (*n* = 5/group). ^*^P < 0.05, ^**^P < 0.01 (Bonferroni test).

Dysfunctional mitochondria can be removed via mitophagy, which is mainly regulated by the PINK1/Parkin signaling pathway [23, 24]. To evaluate whether TP supplementation stimulates mitophagic activity in the aging brain, PINK1, Parkin, and P62 expression levels were analyzed by western blotting in the experimental rat groups. Significant intergroup differences in PINK1, Parkin, and P62 expression were detected in the SN (Figure 11A–11D; PINK1, F(2,12) = 58.001; Parkin, F(2,12) = 44.445; P62, F(2,12) = 81.016; *P* < 0.01) and HIPP (Figure 11E–11H; PINK1, F(2,12) = 102.526; Parkin, F(2,12) = 165.632; P62, F(2,12) = 8.152; *P* < 0.01). PINK1 and Parkin protein levels were reduced in the SN and HIPP of 24Mon rats compared with 6Mon rats, and elevated instead in 24Mon-TP rats relative to 24Mon rats (Figure 11A–11C for SN; Figure 11E–11G for HIPP). Significantly increased P62 expression was in turn detected in the SN and HIPP of 24Mon rats relative to 6Mon rats; such increase was however significantly attenuated in 24Mon-TP rats (Figure 11A and 11D for SN; Figure 11E and 11H for HIPP). These findings suggest that TP supplementation promotes mitophagy in the aged male rat brain by activating PINK1/Parkin signaling.

## DISCUSSION

This study revealed the amelioratory effects of testosterone supplementation on mitochondrial dysfunction in the SN and HIPP of aged male rats. Specifically, we showed that testosterone supplementation attenuated neuronal dysfunction and neuronal loss, decreased MDA content, and alleviated the reductions in the GSH/GSSG ratio and antioxidant capacity in the SN and HIPP of aged male rats, along with improved explorative behavior of them. Moreover, testosterone supplementation significantly improved MMP in the SN and HIPP, enhanced the activity of mitochondrial complexes I and V in the SN and of complexes IV and V in the HIPP, and increased both the expression of PGC-1α, NRF1, and TFAM, and the mitochondrial content in both brain regions. Furthermore, increased PINK1/Parkin and decreased P62 expression suggested that testosterone administration activated mitophagy in the SN and HIPP of aged male rats. These findings strongly suggest that testosterone supplementation ameliorates age-related brain mitochondria dysfunction in male rats by enhancing both mitochondrial antioxidative capacity and mitochondrial biogenesis.

Although aging is an important risk factor for several neurodegenerative diseases, the underlying mechanisms are not fully understood [25]. While different hypotheses have been advanced to explain the aging process, there is growing consensus on the crucial role of mitochondrial dysfunction in age-related cellular dysfunction and disease [26–29]. Functional age-related changes in mitochondria occur in several organs and tissues, including the brain [28, 30]. Accordingly, an increase in ROS production and a reduction in mitochondrial respiratory chain function were observed in the aged brain [27, 28, 31, 32]. In the present study, mitochondrial dysfunction in the aged male rat brain was exemplified by increased MDA levels, decreased GSH/GSSG ratio and MMP, as well as reduced activities of mitochondrial complex I and complex V.

Both physiological (e.g., age-related) and therapeutically induced (e.g., hormone replacement) alterations in circulating hormone levels have shown to influence changes in mitochondrial function [16, 18, 33, 34]. Hormone replacement in adult ovariectomized rats induces beneficial brain mitochondria alterations reflected by a reduction in oxidative stress and endogenous oxidative damage [35]. In turn, orchiectomy was shown to disturb mitochondrial function, evidenced by increased mitochondrial H_2_O_2_ production and decreased MMP in the SN of adult male rats [18]. Moreover, previous studies found that testosterone replacement improves motor behavioral and cognitive deficits [36–39], enhances nigrostriatal dopaminergic activity [38], and reduces dendritic spine loss in hippocampal CA1 pyramidal neurons [37] in aged male rodents. These findings, along with those of the present study, demonstrated that cognitive/behavioral deficits and mitochondrial dysfunction in the aged male brain are, to some extent, related to decreased serum testosterone levels.

The above findings indicate that enhancing the antioxidant capacity of mitochondria and improving mitochondrial function are two important strategies to maintain normal neuronal function during aging. Our study showed that testosterone supplementation increased PGC-1α expression and stimulated the transcription, translation, and functional activity of GSH-PX, CAT, and Mn-SOD in the aged male rat brain. Previous studies showed that PGC-1α promotes mitochondrial biogenesis by coactivating NRF-1 to upregulate TFAM expression [40, 41]. Moreover, PGC-1α exerts also important antioxidative effects by promoting the transcription of ROS-detoxifying enzymes such as GSH-PX, CAT, and Mn-SOD [42]. Accordingly, knockout of PGC-1α in mice dramatically increases sensitivity to oxidative stress-induced damage in SN dopaminergic cells and hippocampal neurons, while increasing PGC-1α levels protects cultured neurons from oxidative stress-mediated death [42]. Stimulating mitochondrial biogenesis is another important strategy to maintain normal mitochondrial function and prevent or attenuate symptoms of common age-related neurodegenerative conditions [43]. Mitochondrial biogenesis entails a complex process by which new mitochondria are formed from preexisting mitochondria through coordinated interactions between nuclear and mitochondrial genes [43, 44]. An *in vitro* study showed that testosterone increases PGC1α, NRF-1, NRF-2, and TFAM transcription in C_2_C_12_ myotubes, and this effect is abolished by an androgen receptor antagonist [45]. Along these lines, orchiectomy was shown to decrease PGC-1α, NRF-1, NRF-2, and TFAM gene expression in the hippocampus of adult rats, while concurrent supplementation with testosterone fully restored their expression [16]. All these data strongly suggest that aging-associated impaired mitochondrial biogenesis results, at least in part, from testosterone deficiency leading to decreased expression of PGC-1α and its downstream effectors. In addition to upregulating PGC-1α, NRF-1, and TFAM expression, testosterone supplementation increased mitochondrial content in the SN and HIPP of aged male rats, as reflected by enhanced CS activity, increased mtDNA copy number, and upregulated mRNA and protein expression of mtDNA-encoded subunits ND1, COX1, and ATP6.

Increased PINK1/Parkin and decreased P62 expression in the SN and HIPP of testosterone-supplemented rats further suggested that enhanced mitophagy likely contributes to the beneficial effect of testosterone supplementation against mitochondrial dysfunction in the aged rat brain. Under stress conditions, PINK1 accumulates in the surface of damaged mitochondria and recruits and phosphorylates Parkin, which undergoes extensive ubiquitination leading to recruitment of autophagy receptors such as P62 to elicit selective degradation of defective mitochondria via mitophagy [46–48]. Thus, although we did not provide direct evidence of enhanced mitophagic activity, the changes observed in PINK1/Parkin and P62 expression allow us to hypothesize that testosterone optimizes the disposal of defective mitochondria during aging to maintain mitochondrial homeostasis in the brain.

Normal mitochondrial function is inextricably linked with proper and sustained activity of oxidative phosphorylation complexes. Decreased activities of mitochondrial complex I and complex IV have been reported in the aging brain [49–52]. Compared to younger rats, in the present study significantly decreased complex I activity was found in the SN, but not in the HIPP, of aged male rats. Indeed, mitochondrial complex I activity in the SN seems to undergo a region-specific, aged-related decline in male rats, probably related to dopamine auto-oxidation in dopaminergic neurons [53]. Previous studies showed that reduced mitochondrial complex I and complex IV activities are characteristic features of common age-related neurodegenerative diseases [54–56]. Accumulation of amyloid β peptide, a hallmark of AD, impairs the activity of mitochondrial complex IV, leading to increased ROS levels and ATP depletion in AD brains [54]. In turn, reduced mitochondrial complex I activity has been reported in affected brain areas of patients with PD [54–56]. Indeed, consistent with our preclinical findings and based on robust clinical evidence, it was hypothesized that the age-related decline in testosterone levels in men may act as a "second hit" to impair neurocognitive function and precipitate neurodegenerative disease [11].

There are some unresolved questions about the present results. First, the molecular mechanisms by which testosterone administration increased PGC-1α expression and antioxidant enzyme activities in the aged rat brain were not specifically explored. Nevertheless, available evidence showed that testosterone increases the expression and phosphorylation of 5’-AMP-activated protein kinase (AMPK, a known PGC-1α activator [57]), in adipose tissue and skeletal muscle of hypogonadism patients [58] and triggers cultured cardiomyocyte hypertrophy through activation of AMPK pathways [59]. We presumed that testosterone increased PGC-1α expression in aged rat brain via AMPK activation. Testosterone-upregulated PGC-1α contributes to increased activities of GSH-PX, CAT and Mn-SOD in TP-supplemented aged rat brain, since PGC-1α activation increases GSH-PX, CAT and Mn-SOD expression [42]. It is known that the main mechanism of testosterone action involves direct binding to the intracellular androgen receptor to induce gene transcription [60]. Whether testosterone directly increased PGC-1α expression in aged rat brain and whether testosterone increases GSH-PX, CAT and Mn-SOD expression/activity without through PGC-1α should be performed through future *in vitro* experiments. Second, based on the previous studies, in the present work we evaluated only a single-dosing regimen of TP [18, 61]. Since TP dosage is an important determinant of its neurological effects [18, 62, 63], the impact of different TP doses on age-related brain mitochondria dysfunction merits further scrutiny. Third, the potential effects of long-term vehicle (sesame oil) administration (used here at 40 μl/kg) in aging rats were not investigated. A recent study showed that sesame oil has potential neuroprotective actions against oxidative damage [64]. Therefore, further analysis is required to discriminate testosterone-specific effects on age-related mitochondria dysfunction in the brain.

In conclusion, our study revealed that testosterone supplementation improved exploratory behavior, attenuated neuronal dysfunction and neuronal loss, and ameliorated mitochondrial dysfunction by enhancing both mitochondrial antioxidative capacity and mitochondrial biogenesis of aged male rats. Our findings may be relevant for the design of testosterone-based therapies aimed at preventing or treating cognitive-behavioral deficits and diseases associated with aging.

## MATERIALS AND METHODS

### Animals

Male Sprague-Dawley rats were supplied by the Experimental Animal Center of Hebei Medical University and housed in a temperature-controlled room (22 ± 2°C) on a 12-h light-dark cycle (lights on at 6:00 AM). Food and water were available *ad libitum*. All the experimental procedures were approved by the Committee of Ethics on Animal Experiments of Hebei Medical University. The rats were divided into three groups: 6-month-old group (6Mon, *n* = 48), control aged group (24Mon, *n* = 48) and TP-supplemented aged group (24Mon-TP, *n* = 48). Based on previous studies [18, 61], rats in the 24Mon-TP group received daily subcutaneous injections of TP (1 mg/kg per day) over 12 weeks starting at 21 months of age. The rats in the 24Mon control group were subjected to a similar injection regimen, but received instead only sesame oil (40 μl/kg).

### Open field test

The open field device consisted of 4 black walls and a white bottom (100 cm × 100 cm × 40 cm) placed in a sound-attenuating chamber and illuminated with a 20 lux light source. Rats were individually placed at the center of the field and their activity documented with a digital video camera for 5 min [38]. Exploratory behavior parameters, i.e., walking, climbing, rearing, and sniffing, were analyzed. The data presented correspond to averaged measurements derived from 2 tests.

### Sample preparation

Rats were sacrificed by decapitation and their brains quickly removed for biochemical assays, MMP detection, quantitative real-time PCR (qPCR), and western blot analysis. The tissue block containing the SN or HIPP was dissected on an ice-cold plate under stereomicroscopy and immediately processed for assays of MDA, GSH, GSH-PX, Mn-SOD, MMP, and mitochondrial complexes activities, or snap-frozen in liquid nitrogen and stored at −80°C for qPCR and western blot assays. For immunohistochemistry (IHC) and hematoxylin-eosin (HE) staining studies, animals were anesthetized and perfused transcardially with 4% paraformaldehyde in 0.1 M phosphate buffer (PB, pH 7.4). Brains were postfixed in the same fixative for 4 h at 4°C. The brain blocks were dehydrated in graded ethanol, cleared in xylene, and embedded in paraffin for IHC and HE analysis.

### Assessment of oxidative stress and antioxidant enzyme activity

SN or HIPP tissue blocks were homogenized with 0.01M ice-cold phosphate buffer saline (PBS, pH7.4) and centrifuged at 14,000 × g for 15 min at 4°C. MDA levels, GSH/GSSG ratio, and GSH-PX, CAT, CuZn-SOD, Mn-SOD, and CS activities were measured spectrophotometrically in the supernatant based on the instructions of the corresponding detection kits (MDA: Code A003-1; GSH/GSSG: Code A061-1; GSH-PX: Code A005-1; CAT: Code A007-1; CuZn-SOD and Mn-SOD: Code A001-2; Nanjing Jiancheng Bioengineering Institute, China. CS: Cat. No. 701040, Cayman, USA).

For detection of enzymatic activities of mitochondrial complexes I, II, III, IV, and V, mitochondria were isolated from SN or HIPP tissue blocks using a Tissue Mitochondria Isolation Kit (Code C3606, Beyotime Institute of Biotechnology, China). In brief, the tissue blocks were homogenized in ice-cold buffer (10 mM HEPES, pH 7.5, containing 200 mM mannitol, 70 mM sucrose, 1.0 mM EGTA, and 2.0 mg/mL bovine serum albumin) and centrifuged at 1000 × *g* at 4°C for 10 min. The supernatant was centrifuged again at 3500 × *g* at 4°C for 10 min to harvest the mitochondrial pellet. The enzymatic activities of mitochondrial complexes I, II, III, IV, and V were measured using spectrophotometric detection kits for complex I (Cat. A089-1-1), complex II (A089-2-1), complex III (A089-3-1), complex IV (A089-4-1) and complex V (A089-5-1; Nanjing Jiancheng Institute of Biotechnology, China) according to manufacturer’s specifications. Mitochondrial complex activity was normalized to the total protein amount (μmol/min/g protein).

### Mitochondrial membrane potential detection

MMP was estimated in dissociated SN/HIPP cells using Rhodamine 123 (Rh123) fluorescence. After addition of PBS solution, SN/HIPP tissue blocks were grinded and then filtered through a nylon mesh screen. The harvested cells were incubated in Rh123 solution (10 μg/mL, Sigma) at 37°C for 30 min, washed, resuspended in 1 mL PBS, and immediately analyzed by flow cytometry. MMP variations were detected by recording changes in Rh123 fluorescence intensity upon excitation/emission at 488/534 nm.

### Quantitative real-time PCR analysis

1 μg of total RNA from each SN/HIPP tissue block was reverse-transcribed using random primers to obtain the first-strand cDNA template. Then, qPCR was performed with 1 μL of cDNA (diluted 1:10), 2 μL of each specific primer, and 2 × All-in-One™ qPCR Mix (GeneCopoeia Inc., USA) in a final volume of 20 μL. PCR was performed as follows: an initial cycle at 95°C for 15 min, followed by 40 cycles at 95°C for 10 s, 60°C for 20 s, and 72°C for 20 s. The melting curves of the PCR products were analyzed to confirm the specificity of amplification. Gene expression was analyzed using glyceraldehyde-3-phosphate dehydrogenase (*GAPDH*) as internal control. For all samples, qPCR was performed in triplicate. Relative quantification was performed using the 2^−ΔΔCt^ method. Primers sets were as follows:

*GSH-PX* (5′-AATCAGTTCGGACATCAGGAG-3′ and 5′-GAAGGTAAAGAGCGGGTGAG-3′), CAT (5′-ACAACTCCCAGAAGCCTAAGAATG-3′ and 5′-GCTTTTCCCTTGGCAGCTATG-3′), *Mn-SOD* (5′-GGACAAACCTGAGCCCTAAG-3′ and 5′-CAAAAGACCCAAAGTCACGC-3′), *PGC-1α* (5′-GTGCAGCCAAGACTCTGTATGG-3′ and 5′-GTCCAGGTCATTCACATCAAGTTC-3′), *NRF-1* (5′-AAAAGGCCTCATGTGTTTGAGT-3′ and 5′-AGGGTGAGATGCAGAGAACAAT-3′), *TFAM* (5′-GAAAGCACAAATCAAGAGGAG-3′ and 5′-CTGCTTTTCATCATGAGACAG-3′), *ND1* (5′-CCTATGAATCCGAGCATCC-3′ and 5′-ATTGCAGGGAAATGTATCA-3′), *COX1* (5′-AGCCGGGGTGTCTTCTATCT-3′ and 5′-AAAGGATTGGGTCTCCACCT-3'), *ATP6* (5′-TACCACTCAGCTATCTATAAACCTAAGCA-3′ and 5′-AGTTTGTGTCGGAAGCCTAGAATT-3′), and *GAPDH* (5′-GACTCTTACCCACGGCAAGTT-3′ and 5′-GGTGATGGGTTTCCCGTTGA-3′).

### Analysis of mtDNA copy number

Total DNA was extracted from SN/HIPP tissue blocks using an Animal Tissue Genomic DNA kit (ZP307-2, ZOMANBIO, China) according to the manufacturer's protocol. Mitochondrial DNA (mtDNA) copy number was determined by quantifying mitochondrial ND1 (mtND1) and nuclear-encoded beta-2-microglobulin (β2MG) gene expression via qPCR. qPCR was carried out with 1 μL of sample DNA (diluted 1:10), 2 μL of each specific primer and 2×All-in-OneTM qPCR Mix (GeneCopoeia Inc., USA) in a final volume of 10 μL. The following primers were used:

mtND1 (5′-GAGCCCTACGAGCCGTTGCC-3′ and 5′-GCGAATGGTCCTGCGGCGTA-3′) and β2MG (5′-GCGTGGGAGGAGCATCAGGG-3′ and 5′-CTCATCACCACCCCGGGGACT-3′). PCR was performed as follows: an initial cycle at 95°C for 15 min, followed by 40 cycles of 95°C for 10 s, 60°C for 20 s, and 72°C for 20 s. The melting curves of the PCR products were analyzed to confirm the specificity of amplification. qPCR was performed in triplicate. Relative mtDNA copy number was calculated by the ratio between the mtND1 and the nuclear β2MG genes using the 2^−ΔΔCt^ method.

### Western blot analysis

SN/HIPP tissue blocks were homogenized in radioimmunoprecipitation assay buffer containing 1% Triton X-100, 0.1% sodium dodecyl sulfate (SDS), 0.5% sodium deoxycholate, and protease inhibitors (100 μg/mL phenylmethanesulfonyl fluoride, 30 μg/mL aprotinin, and 1 mM sodium orthovanadate) and then sonicated for 10 s (4 times). The samples were centrifuged at 12,000 × *g* for 20 min at 4°C and the supernatants collected and centrifuged again as before. About 50 μg of protein from the final supernatant was diluted with 4× sample buffer (50 mM Tris, pH 6.8, 2% SDS, 10% glycerol, 0.1% bromophenol blue, and 5% β-mercaptoethanol) and heated for 10 min at 95°C. The proteins were separated via SDS polyacrylamide gel electrophoresis on a 4–12% gel and then transferred to a PVDF membrane (Millipore). The membrane was incubated for 1 h with 5% dry skim milk in Tris-buffered saline containing 0.05% Tween 20 (TBST, pH 7.6) at room temperature, rinsed three times with TBST, and incubated overnight with mouse anti-TH (1:10,000, Sigma), rabbit anti-DAT (1:1000, Sigma), rabbit anti-GSH-PX (1:1000, ABclonal), rabbit anti-CAT (1:1000, ABclonal), rabbit anti-Mn-SOD (1:1000, HuaBio), rabbit anti-PGC-1α (1:200, ABclonal), rabbit anti-NRF-1 (1:1000, Abclonal), rabbit anti-TFAM (1:1000, GeneTex), rabbit anti-ND1 (1:500, ABclonal), rabbit anti-COX1 (1:500, ABclonal), rabbit anti-ATP6 (1:500, ABclonal), rabbit anti-PINK1 (1:1000, ABclonal), rabbit anti-Parkin (1:1000, HuaBio), rabbit anti-p62 (1:1000, MBL), or rabbit anti-β-actin at 4°C based on the study purposes. After three washes, the membrane was incubated for 1 h with IRDye^®^ 800-conjugated goat anti-rabbit or goat anti-mouse secondary antibodies (1:10000; Rockland). Protein bands were visualized and quantified by densitometry on an Odyssey infrared scanner (LI-COR Biosciences, USA). Values for target proteins were normalized with respect to those of β-actin, used as endogenous control.

### Immunohistochemistry and hematoxylin-eosin staining

Paraffin-embedded brain blocks were sliced into 5 μm coronal sections and processed for IHC (SN blocks) or hematoxylin-eosin staining (HIPP blocks). Following antigen retrieval and inactivation of endogenous peroxidase activity, SN sections were incubated with mouse anti-TH (1:5000, Sigma) or rat anti-DAT (1:1500, Chemicon) at 4°C overnight. After washing, the sections were respectively incubated with biotinylated goat anti-mouse IgG (1:300) or biotinylated goat anti-rat IgG (1:300) antibodies for 2 h at room temperature. Following 1-h incubation at room temperature in horseradish peroxidase-conjugated streptavidin (1:300), the sections were stained for 5 min in a solution containing 0.05% diaminobenzidine and 0.03% H_2_O_2_ in 0.05 M Tris-HCl buffer (pH 7.6). A computer-assisted image analysis system was used to measure AOD for TH and DAT immunoreactivity in the SN and to count the number of HE-stained cells with pyknotic appearance in the hippocampal CA1 region.

### Serum testosterone assay

Rat trunk blood was collected after decapitation and allowed to rest in open microfuge tubes at room temperature for 30 min to allow the blood to coagulate. Serum was harvested by centrifugation and stored at –80°C until the assay. Serum testosterone levels were measured with a testosterone radioimmunoassay kit based on the manufacturer’s protocol (Tianjin Nine Tripods Medical and Bioengineering Co., Ltd. China).

### Statistical analysis

Data are shown as the mean ± standard deviation (SD). Grubb's test was applied to remove outliers. Tests of normality (Kolmogorov-Smirnov test) and homogeneity variance (Levene’s test) were applied to all the data. If both normal distribution and homogeneity of variance were satisfied, one-way ANOVA with post hoc Bonferroni test was used for group comparison. Otherwise, groups were compared with Kruskal-Wallis test and post hoc Mann-Whitney *U* test. *P <* 0.05 was considered significant. For the Mann-Whitney *U* test, Bonferroni correction was applied and the predefined significance level (*P <* 0.05) was reset at *P* < 0.0167. For body weight data, repeated measures of one-way ANOVA were performed and *P <* 0.05 was considered significant. Statistical analyses were done using SPSS 21 software.
